# Simultaneous virus identification and characterization of severe unexplained pneumonia cases using a metagenomics sequencing technique

**DOI:** 10.1007/s11427-016-0244-8

**Published:** 2016-12-02

**Authors:** Xiaohui Zou, Guangpeng Tang, Xiang Zhao, Yan Huang, Tao Chen, Mingyu Lei, Wenbing Chen, Lei Yang, Wenfei Zhu, Li Zhuang, Jing Yang, Zhaomin Feng, Dayan Wang, Dingming Wang, Yuelong Shu

**Affiliations:** 1 National Institute for Viral Disease Control and Prevention, Collaboration Innovation Center for Diagnosis and Treatment of Infectious Diseases, Chinese Center for Disease Control and Prevention; Key Laboratory for Medical Virology, National Health and Family Planning Commission, Beijing, 102206 China; 2 Guizhou Provincial Center for Disease Control and Prevention, Guiyang, 550004 China

**Keywords:** unexplained pneumonia, metagenomics, next-generation sequencing

## Abstract

Many viruses can cause respiratory diseases in humans. Although great advances have been achieved in methods of diagnosis, it remains challenging to identify pathogens in unexplained pneumonia (UP) cases. In this study, we applied next-generation sequencing (NGS) technology and a metagenomic approach to detect and characterize respiratory viruses in UP cases from Guizhou Province, China. A total of 33 oropharyngeal swabs were obtained from hospitalized UP patients and subjected to NGS. An unbiased metagenomic analysis pipeline identified 13 virus species in 16 samples. Human rhinovirus C was the virus most frequently detected and was identified in seven samples. Human measles virus, adenovirus B 55 and coxsackievirus A10 were also identified. Metagenomic sequencing also provided virus genomic sequences, which enabled genotype characterization and phylogenetic analysis. For cases of multiple infection, metagenomic sequencing afforded information regarding the quantity of each virus in the sample, which could be used to evaluate each viruses’ role in the disease. Our study highlights the potential of metagenomic sequencing for pathogen identification in UP cases.

## INTRODUCTION

Viruses are responsible for the majority of human respiratory infections (Rudan et al., 2013), and various respiratory pathogens such as influenza virus, parainfluenza virus, coronavirus, and rhinovirus are frequently identified in clinical samples. However, pathogens remain undetected by routine clinical testing in approximately 30% of respiratory cases (Heikkinen and Järvinen, 2003). Moreover, many novel viral pathogens, such as the SARS-CorV and MERS viruses (Ding et al., 2003; Ishikane et al., 2015), can infect the human respiratory tract and lead to severe respiratory distress. Therefore, active surveillance of the causative agents involved in respiratory cases is also critical for early detection of emerging

†Contributed equally to this work
*Corresponding author (email: cfswdm@gzscdc.org)
**Corresponding author (email: yshu@cnic.org.cn)

diseases.

Owing to its high specificity and sensitivity in clinical diagnosis, sequence-based PCR is currently the routine approach for virus identification. However, PCR-based methods require sequence information for the targeted agents, and a specific assay needs to be developed for each individual pathogen. Furthermore, as only the pathogen that such an assay is designed to detect will be identified, atypical or emerging pathogens may evade detection by PCR-based methods. In contrast, recently developed next-generation sequencing technology (NGS) combined with a highly sensitive random amplification method can provide a comprehensive view of the pathogenic agents in a given sample, making it suitable for identifying novel and unexpected pathogens (Palacios et al., 2008; Zoll et al., 2015). Moreover, metagenomic sequencing could provide detailed genomic information of the pathogens present in samples, which could be used for genotype characterization and phylogenetic analysis. Based on the taxonomic assignment of output sequences, clinicians can further evaluate the individual influence of each pathogen to disease development (Graf et al., 2016). Thus, compared to the positive/negative result provided by routine PCR, such sequence-based data are more informative to clinicians with regard to determination of the real causative agent of a disease.

Since 2004, the Chinese government has implemented a nationwide surveillance network to monitor unexplained pneumonia (UP) cases (Qian et al., 2011). However, the pathogens causing UP are typically difficult to identify using traditional diagnostic tools. Thus, to examine the potential of NGS and a metagenomic approach for pathogen identification and characterization of UP cases, we collected 33 respiratory samples from severe UP cases in Guizhou Province, China. These samples were subjected to linear amplification and deep sequencing for detection of respiratory viruses. Our study shows that a metagenomic approach could be a promising tool for the clinical diagnosis of UP cases.

## RESULTS

### Virus detection by metagenomic sequencing

Of the 33 samples processed for NGS and metagenomic analysis, 16 were positive for respiratory viruses and comprised 13 virus types (Table 1). HRV was the most frequently

**Table 1** Samples with respiratory viruses detected by metagenomic sequencing[a)]

| Sample ID | Total reads | Human reads (%) | Viruses (Virus reads)* | Coverage** (%) |
|---|---|---|---|---|
| CN02 | 3,697,168 | 2.29 | Influenza virus B (10,973) | 24.02 |
| | | | Human papillomavirus type 10 (122) | 4.30 |
| **CN03** | **993,166** | **1.72** | **Human rhinovirus C (789)** | **84.50** |
| **CN07** | **3,885,096** | **0.64** | **Human rhinovirus A (3,270)** | **83.95** |
| CN08 | 1,086,660 | 9.84 | Human rhinovirus C (2,590) | 64.99 |
| **CN09** | **3,620,770** | **63.37** | **Human adenovirus B 55 (395,570)** | **99.38** |
| | | | Human endogenous retrovirus (68) | 14.09 |
| CN10 | 3,511,030 | 50.86 | Human rhinovirus C (7,031) | 43.67 |
| | | | Human endogenous retrovirus (69) | 6.47 |
| CN11 | 3,976,582 | 0.49 | Human coronavirus HKU1 (10) | 2.58 |
| | | | SEN virus (853) | 57.71 |
| **CN12** | **5,370,692** | **1.84** | **Human coxsackievirus A10 (1,519,427)** | **100.00** |
| | | | Human coronavirus OC43 (6.917) | 49.69 |
| | | | Human respiratory syncytial virus B (5,127) | 59.65 |
| CN14 | 3,946,822 | 0.62 | Human respiratory syncytial virus B (6) | 3.27 |
| **CN15** | **3,853,278** | **0.94** | **Human rhinovirus C (14,918)** | **91.56** |
| CN24 | 4,726,106 | 6.64 | Human rhinovirus C (348) | 23.67 |
| **CN28** | **1,353,140** | **15.75** | **Human parainfluenza virus 3 (57,742)** | **94.06** |
| | | | **Human rhinovirus C (431)** | **93.60** |
| **CN31** | **5,056,875** | **46.78** | **Measles virus (1,047,220)** | **100.00** |
| CN32 | 2,727,310 | 56.62 | Human parainfluenza virus 2 (11,102) | 63.95 |
| | | | Human coronavirus 229E (116) | 2.28 |
| | | | TTV-like mini virus (822) | 32.78 |
| **CN34** | **1,388,632** | **16.24** | **Human rhinovirus C (951,947)** | **100.00** |
| | | | Human endogenous retrovirus (32) | 6.77 |
| **CN35** | **1,238,272** | **3.58** | **Human rhinovirus B (2,647)** | **83.79** |

a) *, Viruses potentially causing respiratory diseases are labeled with a shaded background, the number of reads aligned to each virus is shown in parentheses; **, Viruses with whole-genome coverage ⩾80% are highlighted in bold.

detected virus and was found in nine samples, seven of which were the HRV-C genotype. Two samples indicated dual infections, with co-infection by PIV3/HRV-C and PIV2/HCoV-229E in CN28 and CN32, respectively. A triple infection of HCoV-OC43, human coxsackievirus (HCkV), and RSVB was identified in a 1-year-old child (CN12). All of the detected viruses could be genotyped to subtype or serotype based on the genome sequence assembly. A long genome sequence was assembled for most of the viruses, with 10 having ⩾80% genome coverage and a complete genome sequence directly obtained for three. Common respiratory viruses, such as PIV, HCoV, and HRV, were the primary pathogens detected in the UP cases examined. Of interest, the measles virus, which is not included in the routine PCR panel for respiratory pathogens, was also detected by blind analysis in a 1-month-old patient. Other viruses, such as human endogenous retrovirus and human papillomavirus, were also detected in some samples.

### Taxonomic assignment of NGS data

Viruses could be detected from metagenomic sequencing data by blind analysis. Take CN31 as an example, we obtained 5,056,875 reads and assembled 5,149 contigs, 98 of which were aligned to the measles virus, most of which targeted the measles virus strain MVi/Pennsylvania.USA/20.09. Thus, the complete genome of this strain (GenBank: JN635411.1) was downloaded and mapped back to the clean reads; in which 1,047,220 were assigned, corresponding to 38.92% of the total clean reads.

In addition to the reads belonging to measles virus, there are still a large number of clean reads with no information regarding origin. Thus, we aligned all clean reads to the NCBI nt database and then imported the BLAST results into MEGAN software to detail taxonomic assignment. According to the taxon tree, the measles virus received the most reads assigned, followed by *Streptococcus* and *Homo sapiens* (Figure 1A). After reads assigned to "*Homo sapiens*," "Not assigned," and "Low complexity" were filtered out, the data assigned to the measles virus accounted for 68.60% of the total clean reads with a taxon match, followed by 17.46% and 3.74% reads matching to *Streptococcus* and unclassified *Siphoviridae*, respectively (Figure 1B).

### Virus weight evaluation in multiple infections

Metagenomic sequencing provided an opportunity to evaluate the individual influence of each virus in cases of multiple infection. In the triple-infection case CN12, we obtained 5,370,692 reads and assembled 2,416 contigs, of which three,

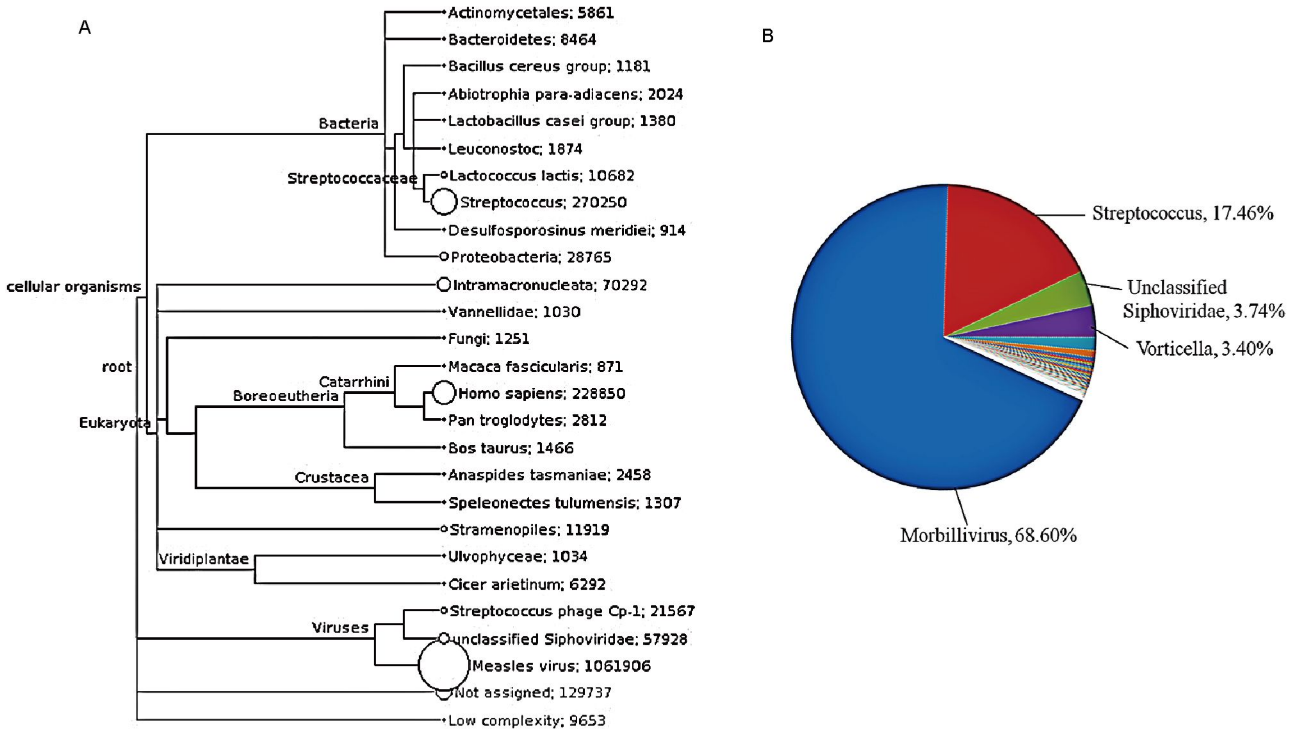

**Figure 1** (Color online) Taxon assignment of metagenomic data from sample CN31. Clean reads were aligned to the NCBI nt database with BLASTn, and the results were imported into Megan software to assign reads to each taxon. A, Read assignment to the phylogenetic tree using Megan. Each circle represents a taxon in the NCBI taxonomy and is scaled logarithmically to indicate the number of reads assigned. Taxa with few reads assigned were collapsed to senior taxa. B, The pie charts correspond to the read distribution among the agents identified at the genus level. Reads matching "*Homo sapiens*," "Not assigned," and "Low complexity" were removed before analysis. The majority of attributable reads were for the measles virus, which belongs to the *Morbillivirus* genus. Genera with <1% reads assigned are not indicated in the pie charts.

25, and 12 were aligned to HCkV, HCoV-OC43, and RSVB, respectively (Table 2). Mapping analysis indicated that 1,519,427, 6,917, and 5,127 reads could be assigned to these three viruses, achieving 100%, 69.5%, and 66.7% virus genome coverage, respectively (Figure 2). Therefore, the reads assigned to HCkV outnumbered those for HCoV-OC43 and RSVB by 200-fold. Virus quantification using digital PCR (dPCR) indicated 1,275 copy μL$^{-1}$ for HCkV in the clinical sample, with 12 copy μL$^{-1}$ for HCoV-OC43 and 6 copy μL$^{-1}$ for RSVB (Table 2). The dPCR results correlated well with the NGS read counts for each virus.

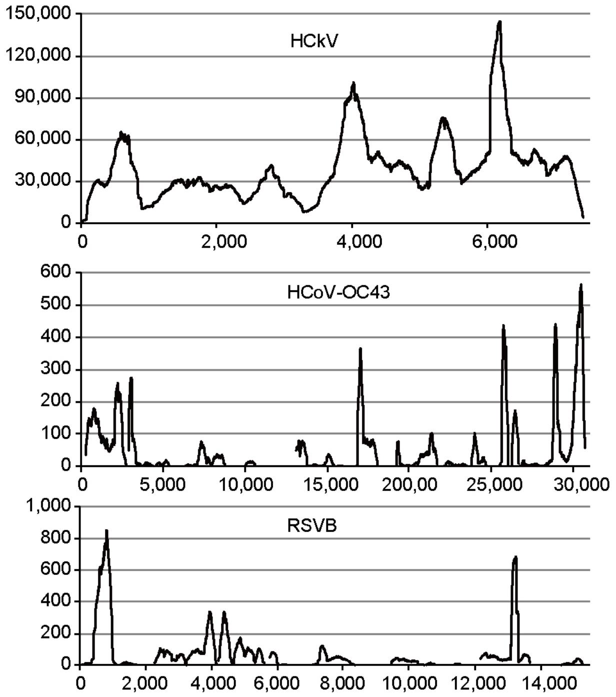

**Figure 2** Genome coverage of the three viruses in triple-infection sample CN12. Three viruses were detected in sample CN12. The reference genome of each virus was identified based on contigs BLAST searches and downloaded for mapping back of the clean reads. The Human coxsackievirus (HCkV) got complete genome covered with high depth, while the human coronavirus OC43 (CorV-OC43) and respiratory syncytial virus B (RSVB), obtained 69.5% and 66.7% of genome covered, respectively. Uncovered regions have no line assignment.

**Table 2** Virus weight evaluation based on NGS reads and digital PCR in the triple-infection sample CN12[a)]

| Virus | HCkV | OC43 | RSVB |
|---|---|---|---|
| Virus load (copies μL$^{-1}$)* | 1,275 | 12 | 6 |
| NO. mapped | 1,519,427 | 6,917 | 5,127 |
| NO. contig** | 3 (3,101) | 25 (1,721) | 12 (1,137) |
| Coverage (%) | 100.0% | 69.5 | 66.7 |
| Total contigs | | 2,416 | |
| Total reads | | 5,370,692 | |

a) *, Virus load was determined by digital PCR; **, NO. of contigs belonging to a specific virus, the number in parentheses indicates the length of the longest contig matching to this virus.

### Phylogenetic analysis of the detected viruses

Phylogenetic information is valuable for determining virus origin, which is critical for management of endemics and outbreaks. For the measles virus in sample CN31, the H gene showed the highest homology to an eastern Chinese strain (MVi/Zhejiang.CHN/10.11/2[H1]) isolated in 2011 (Figure 3A). Phylogenetic analysis including WHO standard strains revealed this measles virus belonged to the H1 genotype (Figure 3A), which predominated the genotype of measles virus in China for 16 years (Zhang et al., 2012). Moreover, this measles virus clustered with the H1a subgroup, which overwhelmed the H1b genotype in China after 2005 (Xu et al., 2014). Sequence alignment including the Chinese vaccine strains Shanghai191 and Changchun47 indicated that the four N-linked glycosylation sites between position 168 and 268 of HA are conserved in our measles virus (Jianzhi and Zhihui, 1983). These glycosylation sites are critical for proper HA protein folding, antigenicity, dimerization, and export from the Golgi (Hu et al., 1994). The Guizhou measles virus strain was also consistent with vaccine strains in the five antigenic epitopes and seven cysteines of HA (Hu et al., 1993); these residues are important for accurate disulfide bonding of monomeric HA glycoproteins (Hu and Norrby, 1994; Plemper et al., 2000).

The complete genome of HCkV in CN05 was directly obtained by metagenomic sequencing, revealing that this virus belongs to genotype A10. The complete genome of this virus showed 99% sequence identity to coxsackievirus A10 strain FY01/AH/CHN/2013 in the NCBI nucleotide database. Phylogenetic analysis based on the partial VP1 sequence showed that this HCkV sequence groups with strains isolated in China after 2009 and that it is distinct from strains isolated in Japan or strains previously isolated in China (Figure 3B). These results indicate that it is a local circulating strain.

## DISCUSSION

The respiratory tract is one of the major interfaces for human contact with external microbes and is thus the target of many viruses, especially RNA viruses (Assiri et al., 2013; Xie et al., 2015). Current molecular tests for respiratory infection are largely pathogen specific, requiring test selection based on the patient's symptoms, which necessitates large panels of expected pathogens and has limited yield. In contrast, NGS-based metagenomic approaches are target independent and provide unbiased identification of common and unexpected pathogens in a given sample. Moreover, metagenomic sequencing assembles genome information directly from clinical samples, allowing assessment of antigenic epitope variation, strain-level typing, and phylogenetic relatedness. In addition, the increasing advances in bench-top sequencers such

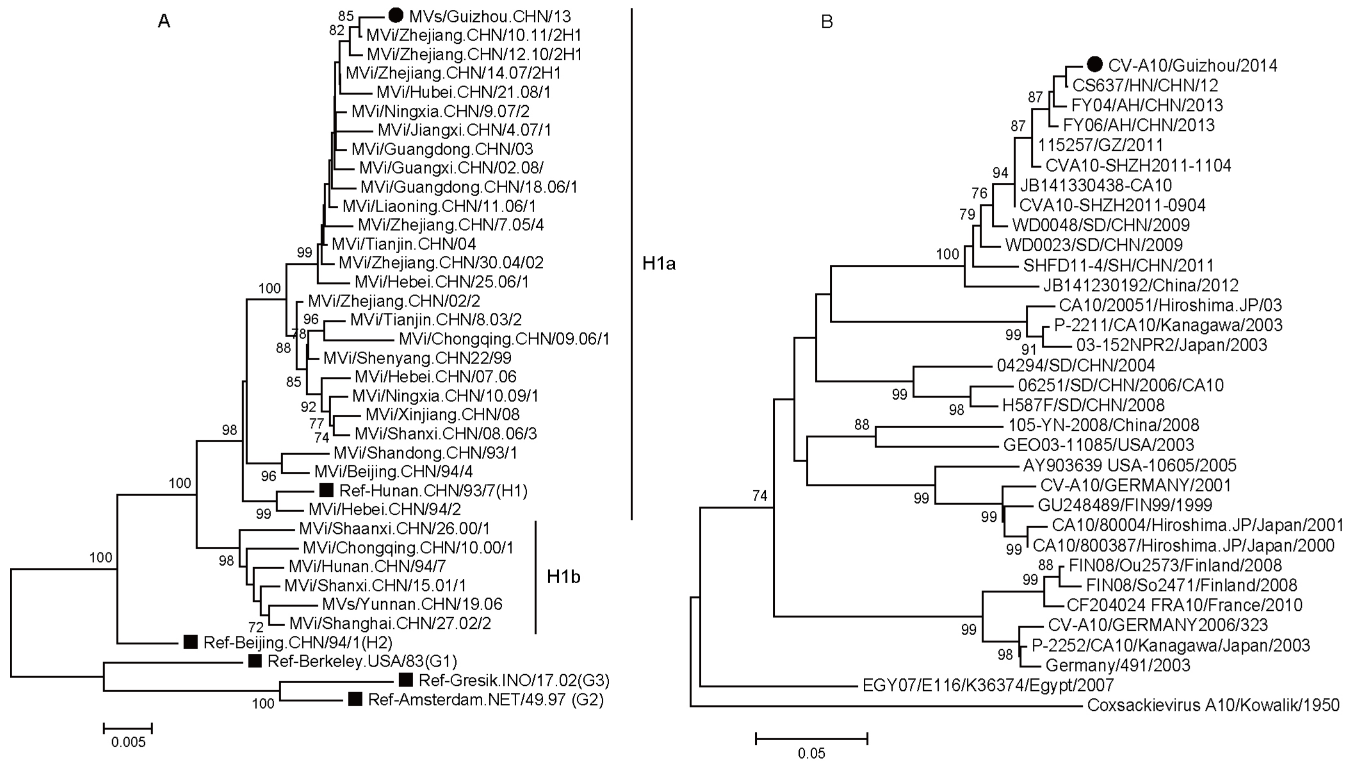

**Figure 3** Phylogenetic analysis of the measles and HCkV viruses. Sequences were aligned with MUSCLE; phylogenetic analysis was performed using Mega 5.1. Bootstrap values <70% are hidden in the trees. A, The complete coding sequence of the hemagglutinin gene was used for phylogenetic analysis of the measles virus in sample CN02. Groups corresponding to cluster H1a and H1b are indicated. The phylogenetic trees are rooted to genotypes G3 and H2, respectively. WHO reference strains are labeled with black squares, and the measles virus we detected is labeled with a black circle. B, Phylogenetic analysis of HCoV-A10 strains based on the partial VP1 gene (269nt). HCoV detected in this study is labeled with a black circle.The results presented here prove that metagenomic sequencing is a promising tool for virus detection and characterization in cases of complex infection. In contrast to routine diagnostic tools, which are designed to detect predefined pathogens, metagenomic sequencing not only enables unbiased pathogen detection but also yields epidemiologically and clinically valuable sequence information. In addition to the decreasing cost of NGS and improved data processing methods, metagenomic sequencing offers unprecedented possibilities for characterizing, diagnosing, and evaluating clinical pathogens in UP cases.

as the Illumina MiSeq and Ion Torrent PGM are associated with an ongoing reduction in cost, making metagenomic sequencing of clinical samples more affordable and available in clinical diagnostic laboratories.

Challenges in pathogen identification often occur for the respiratory infection cases forwarded to CHUPSN, at least with regard to local clinical diagnostic laboratories. Based on the definition of UP cases, viruses should be the main causative agents. Our metagenomic sequencing identified 13 virus types in 16 samples among a total 33 cases tested. All the viruses were identified to the strain or genotype level, as demonstrated by human adenovirus B 55 and HCkV A10. HRV-C was the virus most frequently detected. Although we could not conclude that HRV-C was the causative pathogen of the UP cases because of its high prevalence in non-symptomatic individuals (Briese et al., 2008), attention should be given to HRV-C in young patients, such as the 1-month-old infant case in our study (CN24), as there is evidence that HRV-C may be more virulent in children with pneumonia (Khetsuriani et al., 2008).

Laboratory diagnosis of infectious diseases has historically taken a syndrome-based approach, and the current molecular tests are usually target specific, resulting in challenges when novel or unexpected viruses emerge. Measles is a highly contagious disease characterized by a high fever, cough, and maculopapular rash. Its clinical symptoms are so characteristic that the measles virus is generally not included in common multiple PCR panels, such as the FDA-cleared GenMark eSensor RVP (Pierce and Hodinka, 2012) and CE-IVD-labeled RespiFinder SMART 22 FAST PCR panel (Dabisch-Ruthe et al., 2012), which cover 14 and 22 respiratory pathogens, respectively. The scenario in our study of an infant as young as one month of age infected with measles is a rarity, as protection owing to maternal antibodies usually exists during this early period of life (Kiliç et al., 2003). Thus, identification of the causative agent in such cases would be relatively difficult using routine PCR-based assays. Conversely, our unbiased metagenomic approach directly identified this virus, and we were able to assemble the complete genome sequence, allowing a comprehensive phylogenetic analysis and sequence characterization. Moreover, taxonomic assignment of the total reads indicated that the measles virus accounted for the greatest proportion of NGS output data. Given that no other pathogens were detected in the sample, we are substantially confident that the measles virus was the pathogen causing UP in this infant.

Metagenomics-based pathogen detection is particularly powerful when multiple viruses are detected in a single sample, which could provide a read count-based evaluation of viral load in the sample. For the triple-infection sample CN12, 200-fold more reads were found for HCkV compared to HCoV-OC43 and RSVB. Indeed, the read count ratio of the three virus correlated with the virus burden determined by dPCR (Table 2). A previous study also indicated that viral read counts based on untargeted metagenomics correlated with the viral burden determined by quantitative PCR or the threshold cycle ($C_t$) value provided by Taqman PCR (Graf et al., 2016). Therefore, HCkV appears to have played a predominant role in this patient's UP disease.

Another advantage of metagenomic pathogen identification is the ability to perform pathogen genome sequence assembly from NGS reads, which could be used for phylogenetic analysis and antigenic epitope characterization. Based on the complete genome sequence of the measles virus assembled for CN31, we found that the critical sites in HA responsible for protein folding and antigenic epitopes are conserved between our case and the vaccine strain. The phylogenetic analysis also indicated that this virus belongs to a local circulating lineage. Metagenomic sequencing also assembled the complete genome sequence of HCkV in CN12, and we further determined that it is of the A10 genotype. The HCkV sequence we detected clustered with A10 strains isolated in China in 2013 (Figure 3B), a time when CV-A10 infections were increasing in some regions of the country (Yang et al., 2015). CV-A10 was demonstrated to be associated with severe complications in hand-foot-mouth disease (Lu et al., 2012), which suggests a critical role of this virus in the severe pneumonia of this infant. This molecular epidemiological information based on genome sequence assembly from NGS data can provide valuable characterization of the origin and transmission of infectious diseases (Du et al., 2016).

## MATERIALS AND METHODS

### Sample collection

Samples were collected based on the Chinese Human Unexplained Pneumonia Surveillance Network (CHUPSN), which was established in China in 2004 to address the challenges of the early discovery of novel viruses, such as SARS-CorV. Sentinel hospitals in CHUPSN performed UP case identification and clinical sample collection. According to CHUPSN guidelines, a UP case should be accompanied by fever (>38°C) and pneumonia radiographic results as well as a normal or slightly decreased number of total white blood cells or a decreased lymphocyte differential count in the early stage of the disease. Moreover, no improvement is observed in UP cases after regular antibiotic treatment. After a UP case was defined, an oropharyngeal swab was sampled from the patient and stored in viral transport medium (VTM) at −80°C until testing. The VTM was transferred to the local center for disease control and prevention (CDC) for influenza A (FluA) virus testing by real-time PCR. This study focused on UP patients who developed severe clinical symptoms and required hospitalization in Guizhou Province from 2013 to 2014. Only FluA-negative samples were processed by metagenomic sequencing. A total of 33 patients, 24 males and nine females aged from one month to 82 years, were ultimately enrolled (Table S1 in Supporting Information). All the participants (or guardian for children) provided written informed consent. The collection of clinical samples and implementation of the study were approved by the ethics committee of the Guizhou CDC and National Institute of Viral Disease Control and Prevention, China CDC.

### Sample processing and next-generation sequencing

Clinical specimens for NGS were subjected to a series of pre-treatments before nucleic acid extraction based on the protocol described by Claudia (Kohl et al., 2015). Briefly, 500 μL of VTM was centrifuged at 2,000×*g* for 10 min, and the supernatant was filtered through a 0.22-μm filter (Ultrafree MC, USA) at 2,000×*g* in a microcentrifuge. The filtered VTM was treated with an enzyme cocktail containing 10 U of DNaseI (NEB, USA), 20 U of RNase $I_f$ (NEB), and 10 U of benzonase (Merck, USA) for 2 h to digest naked host nucleic acid. RNA was extracted from the treated sample using an RNeasy mini kit (QIAGEN, Germany) and subjected to linear reverse amplification using the NuGEN Ovation RNA-seq V2 kit (NuGEN, USA). Approximately 100 ng cDNA per sample was used for sequencing with the Ion PGM™ sequencing platform (Thermo Fisher, USA) at a read-length of 200 bp. For samples with scant cDNA produced owing to low viral copies, we used 1 ng of cDNA for library construction with Nextera XT DNA Library Preparation Kit (Illumina, USA) and sequenced the products using the MiSeq (Illumina) platform for 2× 150 cycles with version 2 reagents and the "FASTQ only" workflow.

### Bioinformatic analysis

#### *NGS data processing*

NGS data analysis was primarily conducted using CLC genomic workbench 7.0.5 package. At the filter step, reads with an error rate above 0.05 or a length below 100 bp were trimmed out. Reads passing quality filtering were mapped to human reference genome hg18 with stringent criteria that set the length fraction at 0.9 and the similarity fraction at 0.9 using the CLC mapper software. Unmapped reads were collected as clean reads for downstream analysis.

*Virus identification*

Clean reads were assembled *de novo* with the "*de novo* assembly" tool in the CLC package under the parameter "automatic word size=20, automatic bubble size=50". After assembly and scaffolding, contigs with a length greater than 300 bp were aligned to the NCBI nt database using local BLASTn. The pathogen sequence/genome identified in the BLAST hits was selected as the reference for mapping clean reads under the default parameters in CLC mapper. Consensus sequences were extracted from the mapping results to obtain the targeted virus genome. All clean reads were mapped to the Complete RefSeq release of viral and viroid sequences downloaded from NCBI, which to date contains 6,024 virus reference genome.

*Taxonomic assignment*

Sequence similarity-based taxonomic assignments were applied to non-host data sets. Clean reads were first aligned to the NCBI nt database using local BLASTn with the "-max_target_seqs 5" specification. The BLAST results were then imported into MEGAN software (version 5.10.2) to assign each read to the lowest common ancestor (LCA) of the set of taxa that it hit (Huson et al., 2007). The "min support" (minimum support) parameter was changed to 100 (<0.1% of the total clean reads) to filter out taxa to which only a small number of reads were assigned.

*Phylogenetic analysis*

Phylogenetic analysis of the measles virus was performed based on the complete coding sequence of the surface hemagglutinin (H) protein, and that for enterovirus was based on a partial VP1 sequence. Sequences included in the analysis were aligned using MUSCLE, and a phylogenetic tree was constructed using the neighbor-joining method. The reliability of the phylogenetic inference at each branch node was estimated using the bootstrap method with 1000 replications.

## Digital PCR quantification

Virus quantification was implemented in QuantStudio™ 3D Digital PCR System (Thermo Fisher, USA). Briefly, 5 μL of sample RNA was reverse transcribed to first-strand cDNA using SuperScript VILO cDNA Synthesis Kit (Thermo Fisher). A digital PCR reaction mixture was then prepared with 3.5 μL cDNA product added as the template and loaded onto digital PCR 20K Chips for PCR amplification with GeneAmp® PCR System 9700. The number of virus copies was assessed by the QuantStudio 3D Digital PCR instrument, which imaged the 20K chip and performed a preliminary analysis. Those data in the result meeting all quality thresholds were considered to be valid.

**Compliance and ethics** *The author(s) declare that they have no conflict of interest.*

**Acknowledgements** *We thank Dr. Zhen Zhu in China Institute of Veterinary Drug Control (IVDC) for her help on sequence characterization of the measles virus. This study was supported by the National Mega-projects for Infectious Diseases (2014ZX10004002), and Beijing Municipal Science and Technology Commission grant (D151100002115003).*

Assiri, A., McGeer, A., Perl, T.M., Price, C.S., Al Rabeeah, A.A., Cummings, D.A.T., Alabdullatif, Z.N., Assad, M., Almulhim, A., Makhdoom, H., Madani, H., Alhakeem, R., Al-Tawfiq, J.A., Cotten, M., Watson, S.J., Kellam, P., Zumla, A.I., Memish, Z.A., and Memish, Z.A. (2013). Hospital outbreak of middle east respiratory syndrome coronavirus. N Engl J Med 369, 407–416.

Briese, T., Renwick, N., Venter, M., Jarman, R.G., Ghosh, D., Köndgen, S., Shrestha, S.K., Hoegh, A.M., Casas, I., Adjogoua, E.V., Akoua-Koffi, C., Myint, K.S., Williams, D.T., Chidlow, G., van den Berg, R., Calvo, C., Koch, O., Palacios, G., Kapoor, V., Villari, J., Dominguez, S.R., Holmes, K.V., Harnett, G., Smith, D., Mackenzie, J.S., Ellerbrok, H., Schweiger, B., Schønning, K., Chadha, M.S., Leendertz, F.H., Mishra, A.C., Gibbons, R.V., Holmes, E.C., and Lipkin, W.I. (2008). Global distribution of novel rhinovirus genotype. Emerg Infect Dis 14, 944–947.

Dabisch-Ruthe, M., Vollmer, T., Adams, O., Knabbe, C., and Dreier, J. (2012). Comparison of three multiplex PCR assays for the detection of respiratory viral infections: evaluation of xTAG respiratory virus panel fast assay, RespiFinder 19 assay and RespiFinder SMART 22 assay. BMC Infect Dis 12, 163.

Ding, Y., Wang, H., Shen, H., Li, Z., Geng, J., Han, H., Cai, J., Li, X., Kang, W., Weng, D., Lu, Y., Wu, D., He, L., and Yao, K. (2003). The clinical pathology of severe acute respiratory syndrome (SARS): a report from China. J Pathol 200, 282–289.

Du, J., Yang, L., Ren, X., Zhang, J., Dong, J., Sun, L., Zhu, Y., Yang, F., Zhang, S., Wu, Z., and Jin, Q. (2016). Genetic diversity of coronaviruses in *Miniopterus fuliginosus* bats. Sci China Life Sci 59, 604–614.

Graf, E.H., Simmon, K.E., Tardif, K.D., Hymas, W., Flygare, S., Eilbeck, K., Yandell, M., and Schlaberg, R. (2016). Unbiased detection of respiratory viruses by use of RNA sequencing-based metagenomics: a systematic comparison to a commercial PCR panel. J Clin Microbiol 54, 1000–1007.

Heikkinen, T., and Järvinen, A. (2003). The common cold. Lancet 361, 51–59.

Hu, A., Cattaneo, R., Schwartz, S., and Norrby, E. (1994). Role of N-linked oligosaccharide chains in the processing and antigenicity of measles virus haemagglutinin protein. J Gen Virol 75, 1043–1052.

Hu, A., and Norrby, E. (1994). Role of individual cysteine residues in the processing and antigenicity of the measles virus haemagglutinin protein. J Gen Virol 75, 2173–2181.

Hu, A., Sheshberadaran, H., Norrby, E., and Kövamees, J. (1993). Molecular characterization of epitopes on the measles virus hemagglutinin protein. Virology 192, 351–354.

Huson, D.H., Auch, A.F., Qi, J., and Schuster, S.C. (2007). MEGAN analysis of metagenomic data. Genome Res 17, 377–386.

Ishikane, M., Kato, H., Kawabata, K., Ito, H., Kanayama, A., Matsui, T., and Oishi, K. (2015). The Cutting-edge of Medicine; A recent trend of emerging infections: MERS and Avian influenza A (H7N9). Nihon Naika Gakkai Zasshi 104, 114–119.

Jianzhi, X., and Zhihui, C. (1983). Measles vaccine in the People's Republic of China. Clin Infect Dis 5, 506–510.

Khetsuriani, N., Lu, X., Teague, W.G., Kazerouni, N., Anderson, L.J., and Erdman, D.D. (2008). Novel human rhinoviruses and exacerbation of asthma in children1. Emerg Infect Dis 14, 1793–1796.

Kiliç, A., Altinkaynak, S., Ertekin, V., and Inandi, T. (2003). The duration of maternal measles antibodies in Children. J Trop Pediatr 49, 302–305.

Kohl, C., Brinkmann, A., Dabrowski, P.W., Radonić, A., Nitsche, A., and Kurth, A. (2015). Protocol for metagenomic virus detection in clinical specimens. Emerg Infect Dis 21, 48.

Lu, Q.B., Zhang, X.A., Wo, Y., Xu, H.M., Li, X.J., Wang, X.J., Ding, S.J.,

Chen, X.D., He, C., Liu, L.J., Li, H., Yang, H., Li, T.Y., Liu, W., and Cao, W.C. (2012). Circulation of coxsackievirus A10 and A6 in hand-foot-mouth disease in China, 2009–2011. PLoS ONE 7, e52073.

Palacios, G., Druce, J., Du, L., Tran, T., Birch, C., Briese, T., Conlan, S., Quan, P.L., Hui, J., Marshall, J., Simons, J.F., Egholm, M., Paddock, C.D., Shieh, W.J., Goldsmith, C.S., Zaki, S.R., Catton, M., and Lipkin, W.I. (2008). A new arenavirus in a cluster of fatal transplant-associated diseases. N Engl J Med 358, 991–998.

Pierce, V.M., and Hodinka, R.L. (2012). Comparison of the GenMark Diagnostics eSensor respiratory viral panel to real-time PCR for detection of respiratory viruses in children. J Clin Microbiol 50, 3458–3465.

Plemper, R.K., Hammond, A.L., and Cattaneo, R. (2000). Characterization of a region of the measles virus hemagglutinin sufficient for its dimerization. J Virol 74, 6485–6493.

Qian, Y.H., Su, J., Shi, P., He, E.Q., Shao, J., Sun, N., Zu, R.Q., and Yu, R.B. (2011). Attempted early detection of influenza A (H1N1) pandemic with surveillance data of influenza-like illness and unexplained pneumonia. Influenza Other Respir Viruses 5, e479–e486.

Rudan, I., O'Brien, K.L., Nair, H., Liu, L., Theodoratou, E., Qazi, S., Luksic, I., Fischer Walker, C.L., Black, R.E., Campbell, H., and Child Health Epidemiology Reference, G. (2013). Epidemiology and etiology of childhood pneumonia in 2010: estimates of incidence, severe morbidity, mortality, underlying risk factors and causative pathogens for 192 countries. J Glob Health 3, 010401.

Xie, Q., Cao, Y.J., Su, J., Wu, X.B., Wan, C.S., Ke, C.W., Zhao, W., and Zhang, B. (2015). Genomic sequencing and analysis of the first imported Middle East Respiratory Syndrome Coronavirus (MERS CoV) in China. Sci China Life Sci 58, 818–820.

Xu, S., Zhang, Y., Rivailler, P., Wang, H., Ji, Y., Zhen, Z., Mao, N., Li, C., Bellini, W.J., Xu, W., and Rota, P.A. (2014). Evolutionary genetics of genotype H1 measles viruses in China from 1993 to 2012. J Gen Virol 95, 1892–1899.

Yang, Q., Ding, J., Cao, J., Huang, Q., Hong, C., and Yang, B. (2015). Epidemiological and etiological characteristics of hand, foot, and mouth disease in Wuhan, China from 2012 to 2013: Outbreaks of Coxsackieviruses A10. J Med Virol 87, 954–960.

Zhang, Y., Xu, S., Wang, H., Zhu, Z., Ji, Y., Liu, C., Zhang, X., Sun, L., Zhou, J., Lu, P., Hu, Y., Feng, D., Zhang, Z., Wang, C., Fang, X., Zheng, H., Liu, L., Sun, X., Tang, W., Wang, Y., Liu, Y., Gao, H., Tian, H., Ma, J., Gu, S., Wang, S., Feng, Y., Bo, F., Liu, J., Si, Y., Zhou, S., Ma, Y., Wu, S., Zhou, S., Li, F., Ding, Z., Yang, Z., Rota, P.A., Featherstone, D., Jee, Y., Bellini, W.J., and Xu, W. (2012). Single endemic genotype of measles virus continuously circulating in China for at least 16 years. PLoS ONE 7, e34401.

Zoll, J., Rahamat-Langendoen, J., Ahout, I., de Jonge, M.I., Jans, J., Huijnen, M.A., Ferwerda, G., Warris, A., and Melchers, W.J.G. (2015). Direct multiplexed whole genome sequencing of respiratory tract samples reveals full viral genomic information. J Clin Virol 66, 6–11.

**Open Access** This article is distributed under the terms of the Creative Commons Attribution License which permits any use, distribution, and reproduction in any medium, provided the original author(s) and source are credited.

## SUPPORTING INFORMATION

**Table S1** UP Sample Information

The supporting information is available online at life.scichina.com and www.springerlink.com. The supporting materials are published as submitted, without typesetting or editing. The responsibility for scientific accuracy and content remains entirely with the authors.
